# Supplementary material for: Data driven healthcare insurance system using machine learning and blockchain technologies
Source: PeerJ Comput Sci. 2025 Jul 30;11:e2980. doi: 10.7717/peerj-cs.2980 (PMC12453831; doi:10.7717/peerj-cs.2980)
Supplement: Supplemental Information 3 [file peerj-cs-11-2980-s003.zip › cs-106973-Project_code_updated/supplemental/cs-106973-Project_code/Project code/try1/maps/templates/maps/contact1.html]

{% block content %}


Home

Find a Doctor

Generalized Recommendations
Personalized Recommendations

Hospitals
Contact Us
Login
Signup

  
  
  
  

Address

Fatima Jinnah

Women University, Rawalpindi

Phone

+92000 0000000

+92000 1111111

Email

insurancefjwu@gmail.com

Contact Us

  

For all the enquiries, please email us in the form below.

{% csrf\_token %}

Submit

{% endblock content %}
