# Supplementary material for: Data driven healthcare insurance system using machine learning and blockchain technologies
Source: PeerJ Comput Sci. 2025 Jul 30;11:e2980. doi: 10.7717/peerj-cs.2980 (PMC12453831; doi:10.7717/peerj-cs.2980)
Supplement: Supplemental Information 3 [file peerj-cs-11-2980-s003.zip › cs-106973-Project_code_updated/supplemental/cs-106973-Project_code/Project code/try1/maps/templates/maps/doctors.html]

Document


Home
Doctors
Hospitals
Contact Us
Login
Signup

  
  
  
  

# Find the best Doctors

{% csrf\_token %}
{% csrf\_token %}
  
  
  
  

## Select a Department Please:


--Search for Doctor--
{% for department in departments %}
{{ department }}
{% endfor %}
  
Find Doctors


{% csrf\_token %}

## Select a Department Please:


--Search for Doctor--
{% for department in departments %}
{{ department }}
{% endfor %}
  
Submit

{% if recommended\_doctors %}

## Recommended Doctors:

{% for doctor in recommended\_doctors %}
{% endfor %}

{% endif %}

{% block content %}
{% csrf\_token %}

{% if recommended\_doctors is not empty %}

{% for doctor in recommended\_doctors %}

**{{ doctor.doctor\_name }}**

{{ doctor.hospital\_name }}

{{ doctor.department }}

  

Ratings:

{{ doctor.Reviews }}

Fee:

{{ doctor.fee }}

{% endfor %}

{% endif %}
]
{% endblock %}
