# Supplementary material for: Data driven healthcare insurance system using machine learning and blockchain technologies
Source: PeerJ Comput Sci. 2025 Jul 30;11:e2980. doi: 10.7717/peerj-cs.2980 (PMC12453831; doi:10.7717/peerj-cs.2980)
Supplement: Supplemental Information 3 [file peerj-cs-11-2980-s003.zip › cs-106973-Project_code_updated/supplemental/cs-106973-Project_code/Project code/try1/maps/templates/maps/footer.html]

Document


  
  
  


#### company

- About us
- Our Services
- Privacy Policy
- Sign up

#### get help

- Contact Us
- FAQ

#### Hospitals

- CMH
- MH
- AFIC
- AL-SHIFA

#### follow us
