# Supplementary material for: Data driven healthcare insurance system using machine learning and blockchain technologies
Source: PeerJ Comput Sci. 2025 Jul 30;11:e2980. doi: 10.7717/peerj-cs.2980 (PMC12453831; doi:10.7717/peerj-cs.2980)
Supplement: Supplemental Information 3 [file peerj-cs-11-2980-s003.zip › cs-106973-Project_code_updated/supplemental/cs-106973-Project_code/Project code/try1/maps/templates/maps/hospitals.html]

Document


Home

Find a Doctor

Generalized Recommendations
Personalized Recommendations

Hospitals
Contact Us
Login
Signup

  
  
  


These hospitals are on the panel of Fatima Jinnah Women University which include **Armed Forces Institute of
Cardiology** , **Combined Military Hospital**, **Shifa International Hospital**,
**Military Hospital**. Fatima Jinnah Women
University provides health services to its employees through these hospitals.

## Shifa International Hospital, Islamabad

The Hospital was incorporated on September 20, 1987 as a
Private Limited Company and converted into Public Limited Company on October 12, 1989.
The idea of developing a high class medical facility in Pakistan was conceived in New York, USA in the mid of 1985.
The construction activity to prepare the site for laying the foundation of the Hospital
was started in the beginning of 1988. After extensive discussions and many meetings,
the present site in the capital city of Islamabad was selected for this project. The name Shifa was approved because of its
comprehensiveness, originating from our culture, belief and values in totality.
It was the vision of the founding Sponsors which took its shape into reality.
Allah’s grace made it feasible and viable to carry out such a gigantic and enormous venture.

## Combined Military Hospital, Rawalpindi

The hospital was established in 1857 as a small military
hospital for the British army during the colonial period.
Over the years, it grew in size and scope and became a major healthcare facility for the region.
During World War II, the hospital played a vital role in treating wounded soldiers
from both the Allied and Axis powers. After the partition of India in 1947,
the hospital was taken over by the newly formed Pakistan army and renamed as
Combined Military Hospital (CMH).
The Combined Military Hospital Rawalpindi is a tertiary care military hospital in Rawalpindi.
It is headed by a Major General from the Army Medical Corps (Pakistan).
It provides specialized treatment to the armed forces personnel, their immediate families as well as civilians.

## Military Hospital, Rawalpindi

The hospital was established in 1887 as "Indian Troops Hospital".It was a 50 bedded facility for Indian military troops.
At the time of independence the hospital grew to 200 beds and was designated as the "Military Hospital".
The Pak Emirates Military Hospital Rawalpindi is the largest hospital of the Pakistan Armed Forces,
being one of the hospitals in the Pakistan Army with an ISO certification, located in the city of Rawalpindi.
Hospital was renamed as "Pak Emirates Military Hospital" in July 2018 after a grant of $108 million
given by the Government of United Arab Emirates as part of United Arab Emirates Pakistan Assistance Program.

## AFIC, Rawalpindi

The Armed Forces Institute of Cardiology also known as the National Institute of Heart Diseases
or NIHD is a government and military cardiac hospital located in Rawalpindi Cantonment,
Punjab, Pakistan. The Pakistan Army Cardiac Service started in 1953 in Cardiothoracic Centre Rawalpindi.
The first angiography was done in 1969 and the first open heart surgery was done in 1970.
In 1978, the Cardiothoracic Centre Rawalpindi was upgraded as Armed Forces Institute of
Cardiology. This 800-bed cardiac health care institute is a major institute and
hospital in Pakistan.The hospital delivers heart disease and health care services
to people of Pakistan Armed Forces and fellow citizens of Pakistan.

  
  
  


#### company

- About us
- Our Services
- Privacy Policy
- Sign up

#### get help

- Contact Us
- FAQ

#### Hospitals

- CMH
- MH
- AFIC
- AL-SHIFA

#### follow us
