# Supplementary material for: Data driven healthcare insurance system using machine learning and blockchain technologies
Source: PeerJ Comput Sci. 2025 Jul 30;11:e2980. doi: 10.7717/peerj-cs.2980 (PMC12453831; doi:10.7717/peerj-cs.2980)
Supplement: Supplemental Information 3 [file peerj-cs-11-2980-s003.zip › cs-106973-Project_code_updated/supplemental/cs-106973-Project_code/Project code/try1/maps/templates/maps/login.html]

Login


Home

Find a Doctor

Generalized Recommendations
Personalized Recommendations

Hospitals
Contact Us
Login
Signup

## Login

{% csrf\_token %}
{% if error\_message %}

{{ error\_message }}

{% endif %}
Username:
  
Password:
  
 Login
