# Supplementary material for: Data driven healthcare insurance system using machine learning and blockchain technologies
Source: PeerJ Comput Sci. 2025 Jul 30;11:e2980. doi: 10.7717/peerj-cs.2980 (PMC12453831; doi:10.7717/peerj-cs.2980)
Supplement: Supplemental Information 3 [file peerj-cs-11-2980-s003.zip › cs-106973-Project_code_updated/supplemental/cs-106973-Project_code/Project code/try1/maps/templates/maps/maps.html]

Home


Home

Find a Doctor

Generalized Recommendations
Personalized Recommendations

Hospitals
Contact Us
Login
Signup

  
  
  
  
  
  

## We recommend the care you need. We bring the compassion you want

I want to find a doctor
I want to register for the insurance

  


|  |  |
| --- | --- |
| **Hospital Name** | {{ map | safe }} |
| **COMBINED MILITARY HOSPITAL** |
| **SHIFA INTERNATIONAL HOSPITAL** |
| **MILITARY HOSPITAL** |
| **ARMED FORCES INSTITUTE OF CARDIOLOGY** |

  
  

## Find doctors by health concern

|  |  |  |  |  |  |  |  |  |  |  |  |  |  |  |  |  |  |
| --- | --- | --- | --- | --- | --- | --- | --- | --- | --- | --- | --- | --- | --- | --- | --- | --- | --- |
|  |  |  |  |  |  |  |  |  || Eye Specialist | Neurologist | Child Specialist | Cardiologist | ENT Specialist | Dentist | Skin Specialist | Orthopedic Surgeon | Gynecologist |

|  |  |  |  |
| --- | --- | --- | --- |
| **Hospital Names **Location** | **Fee** | **Distance** |** |
| **COMBINED MILITARY HOSPITAL** | H2JW+9V8, CMH Rd, Rawalpindi, Punjab 46000 | 1000-1500 | / |
| **SHIFA INTERNATIONAL HOSPITAL** | Pitras Bukhari Road, Sector H-8/4, Islamabad | 3300 |  |
| **MILITARY HOSPITAL** | Abid Majeed Rd, Rawalpindi. | 1000-1500 |  |
| **ARMED FORCES INSTITUTE OF CARDIOLOGY** | Military Hospital Rd, Rawalpindi, Punjab 46000 | 1000-2000 |  |

## Our Objective

- ## 01

  ### Objective 1

  Data Privacy and  
   Security

  Read more
- ## 02

  ### Objective 2

  Reliable Customer Support

  Read more
- ## 03

  ### Objective 3

  Automated Insurance Claims

  Read more
- ## 04

  ### Objective 4

  Personalized Recommendations

  Read more

| COMBINED MILITARY HOSPITAL | SHIFA INTERNATIONAL HOSPITAL | MILITARY HOSPITAL | ARMED FORCES INSTITUTE OF CARDIOLOGY |
| --- | --- | --- | --- |
| - Dermatologist - Gynecologist - Urologistt - Dentist - Oncologist - Child Specialist - Orthopedic Surgeons - Eye Specialist - ENT Specialist - Cardiologist - Neurologist - Nephrologist - Pulmonologist - Gastroenterologist |

 - Dermatologist - Gynecologist - Urologistt - Dentist - Oncologist - Child Specialist - Orthopedic Surgeons - Eye Specialist - ENT Specialist - Cardiologist - Neurologist - Nephrologist - Pulmonologist - Gastroenterologist | - Dermatologist - Gynecologist - Urologistt - Dentist - Oncologist - Child Specialist - Orthopedic Surgeons - Eye Specialist - ENT Specialist - Cardiologist - Neurologist - Nephrologist - Pulmonologist - Gastroenterologist | - Dermatologist - Gynecologist - Urologistt - Dentist - Oncologist - Child Specialist - Orthopedic Surgeons - Eye Specialist - ENT Specialist - Cardiologist - Neurologist - Nephrologist - Pulmonologist - Gastroenterologist |


  
  
  


#### company

- About us
- Our Services
- Privacy Policy
- Sign up

#### get help

- Contact Us
- FAQ

#### Hospitals

- CMH
- MH
- AFIC
- AL-SHIFA

#### follow us
