# Supplementary material for: Data driven healthcare insurance system using machine learning and blockchain technologies
Source: PeerJ Comput Sci. 2025 Jul 30;11:e2980. doi: 10.7717/peerj-cs.2980 (PMC12453831; doi:10.7717/peerj-cs.2980)
Supplement: Supplemental Information 3 [file peerj-cs-11-2980-s003.zip › cs-106973-Project_code_updated/supplemental/cs-106973-Project_code/Project code/try1/media/deptvsdiv.html]

Gynaecology

Paediatrics

Child Specialist

Neurology

Urology

Oncology

Orthopaedics

Cardiology

Nephrology

Pulmonology

Gastroentrology

Dentistry

View More
  
  
  

Endocrinology

Opthalmology

Pathology

Rheumatology

Liver Transplant

General Surgery

Plastic Surgery

Neuro and Thoracic Surgery

Dermatology

General Medicine

Anesthesiology

Audiology

Allergy and Immunology

Emergency

Family Health

Infectious Diseases

Bone Marrow Transplant

Psychiatry

Vascular and Spine Surgery

Pain Management
