# Supplementary figures and images for: Data driven healthcare insurance system using machine learning and blockchain technologies
Source: PeerJ Comput Sci. 2025 Jul 30;11:e2980. doi: 10.7717/peerj-cs.2980 (PMC12453831; doi:10.7717/peerj-cs.2980)

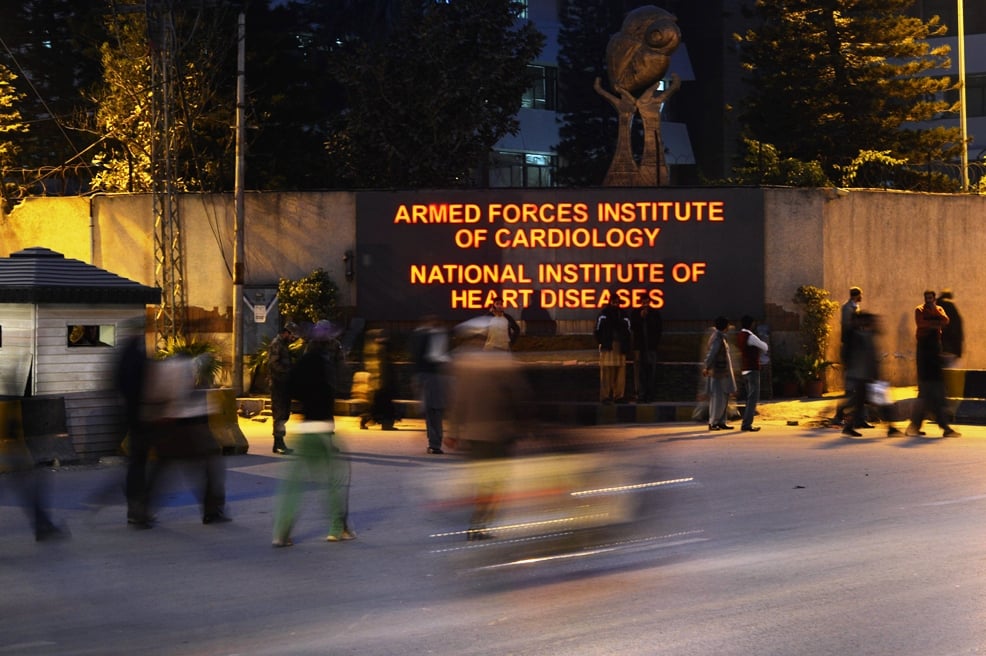

Supplement: Supplemental Information 3 [file peerj-cs-11-2980-s003.zip › cs-106973-Project_code_updated/supplemental/cs-106973-Project_code/Project code/try1/media/afic.jpg]

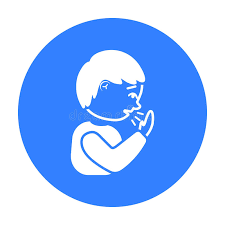

Supplement: Supplemental Information 3 [file peerj-cs-11-2980-s003.zip › cs-106973-Project_code_updated/supplemental/cs-106973-Project_code/Project code/try1/media/all.jpg]

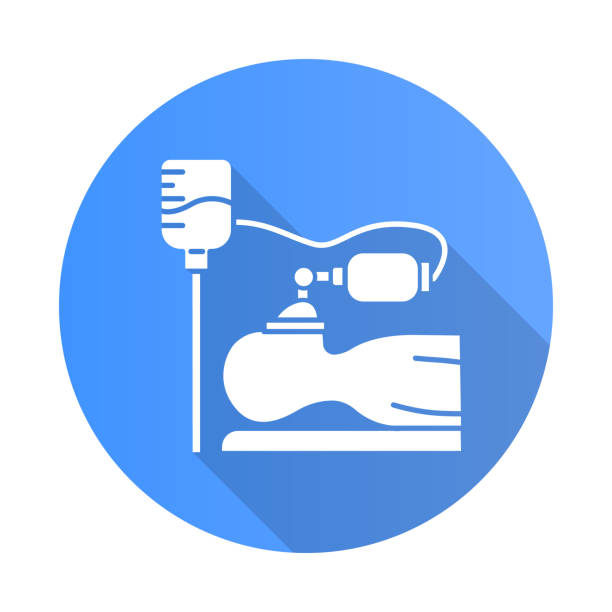

Supplement: Supplemental Information 3 [file peerj-cs-11-2980-s003.zip › cs-106973-Project_code_updated/supplemental/cs-106973-Project_code/Project code/try1/media/anes.jpg]

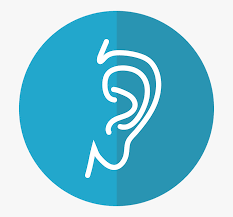

Supplement: Supplemental Information 3 [file peerj-cs-11-2980-s003.zip › cs-106973-Project_code_updated/supplemental/cs-106973-Project_code/Project code/try1/media/audio.jpg]

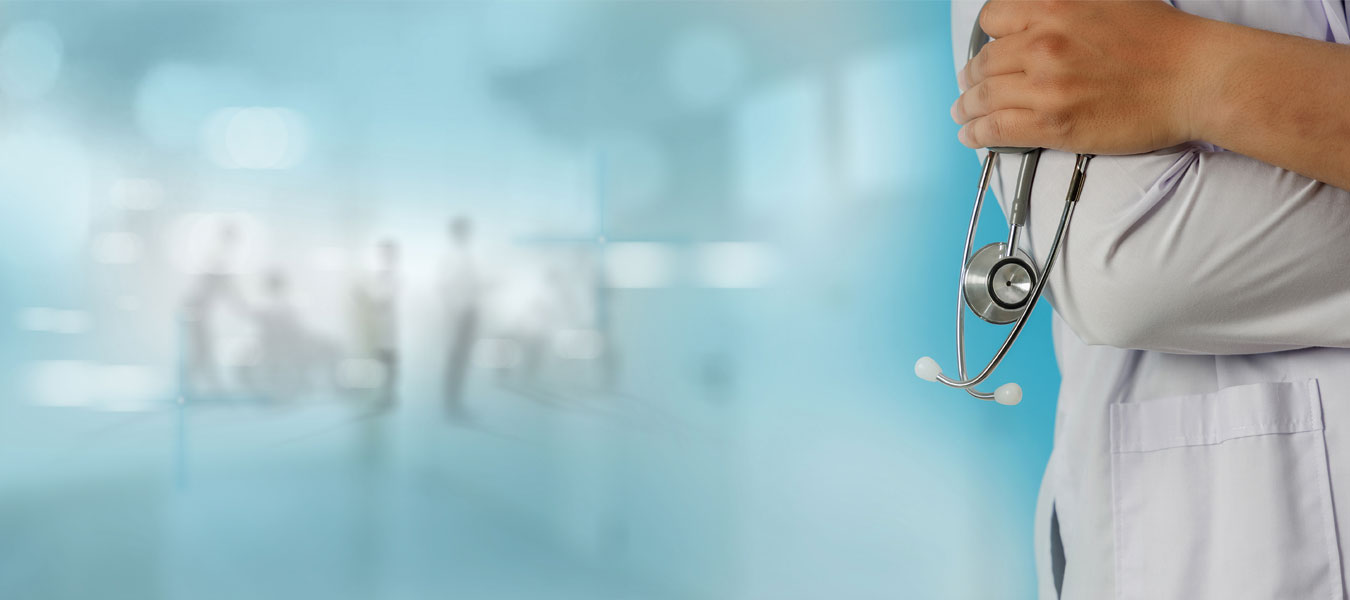

Supplement: Supplemental Information 3 [file peerj-cs-11-2980-s003.zip › cs-106973-Project_code_updated/supplemental/cs-106973-Project_code/Project code/try1/media/banner3.jpg]

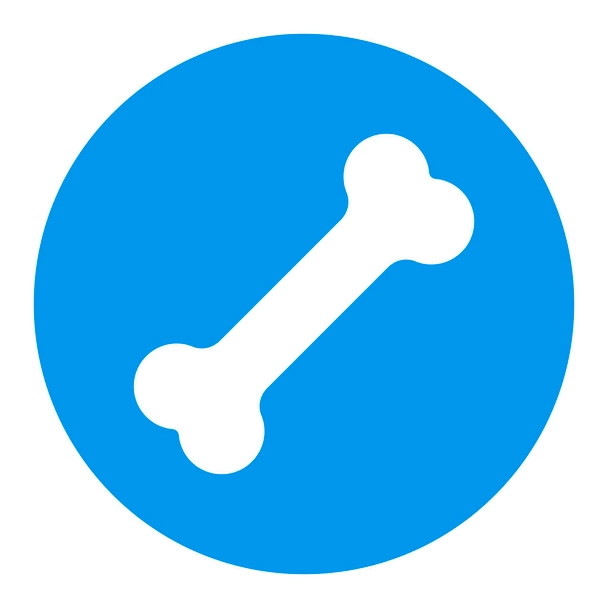

Supplement: Supplemental Information 3 [file peerj-cs-11-2980-s003.zip › cs-106973-Project_code_updated/supplemental/cs-106973-Project_code/Project code/try1/media/bm.jpg]

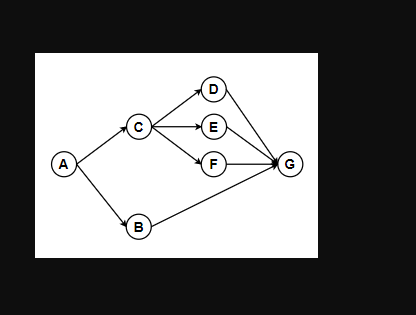

Supplement: Supplemental Information 3 [file peerj-cs-11-2980-s003.zip › cs-106973-Project_code_updated/supplemental/cs-106973-Project_code/Project code/try1/media/Capture1.PNG]

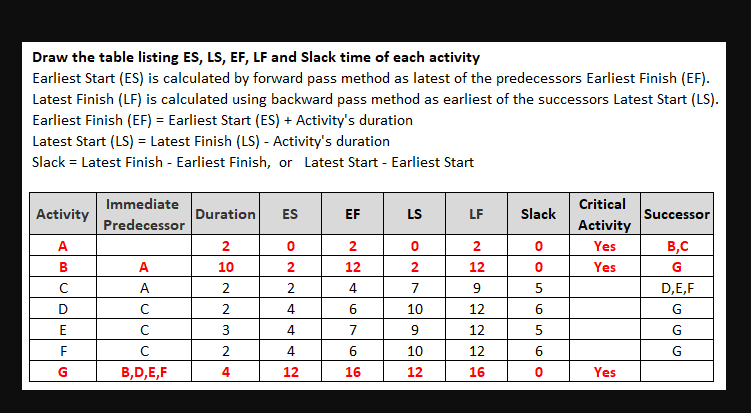

Supplement: Supplemental Information 3 [file peerj-cs-11-2980-s003.zip › cs-106973-Project_code_updated/supplemental/cs-106973-Project_code/Project code/try1/media/Capture2.PNG]

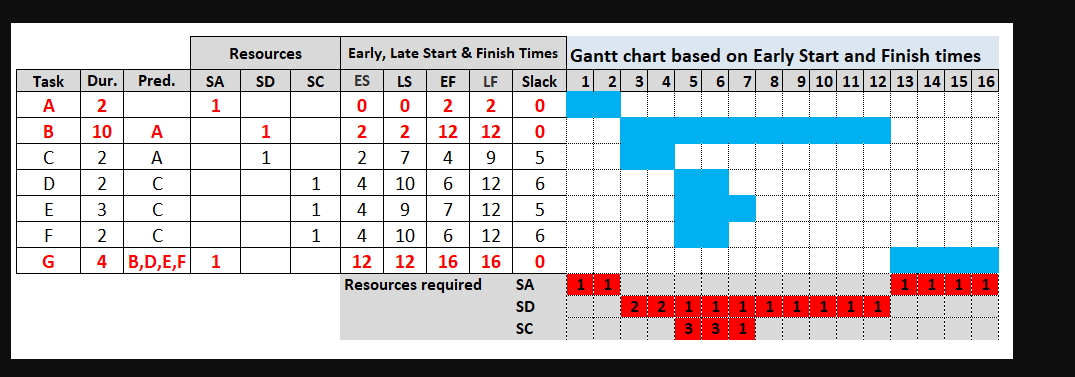

Supplement: Supplemental Information 3 [file peerj-cs-11-2980-s003.zip › cs-106973-Project_code_updated/supplemental/cs-106973-Project_code/Project code/try1/media/Capture3.PNG]

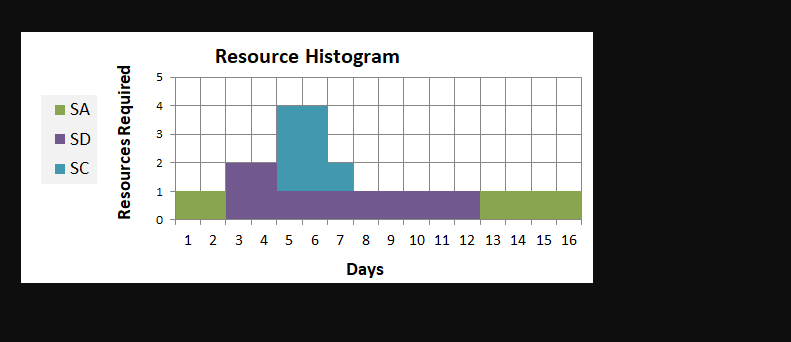

Supplement: Supplemental Information 3 [file peerj-cs-11-2980-s003.zip › cs-106973-Project_code_updated/supplemental/cs-106973-Project_code/Project code/try1/media/Capture4.PNG]

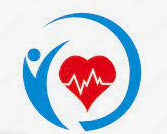

Supplement: Supplemental Information 3 [file peerj-cs-11-2980-s003.zip › cs-106973-Project_code_updated/supplemental/cs-106973-Project_code/Project code/try1/media/card.PNG]

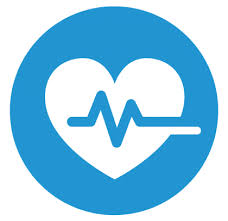

Supplement: Supplemental Information 3 [file peerj-cs-11-2980-s003.zip › cs-106973-Project_code_updated/supplemental/cs-106973-Project_code/Project code/try1/media/cardiology.jpg]

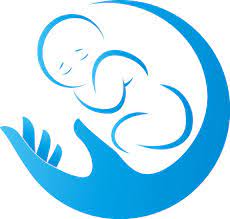

Supplement: Supplemental Information 3 [file peerj-cs-11-2980-s003.zip › cs-106973-Project_code_updated/supplemental/cs-106973-Project_code/Project code/try1/media/child.jpg]

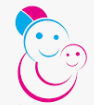

Supplement: Supplemental Information 3 [file peerj-cs-11-2980-s003.zip › cs-106973-Project_code_updated/supplemental/cs-106973-Project_code/Project code/try1/media/child.PNG]

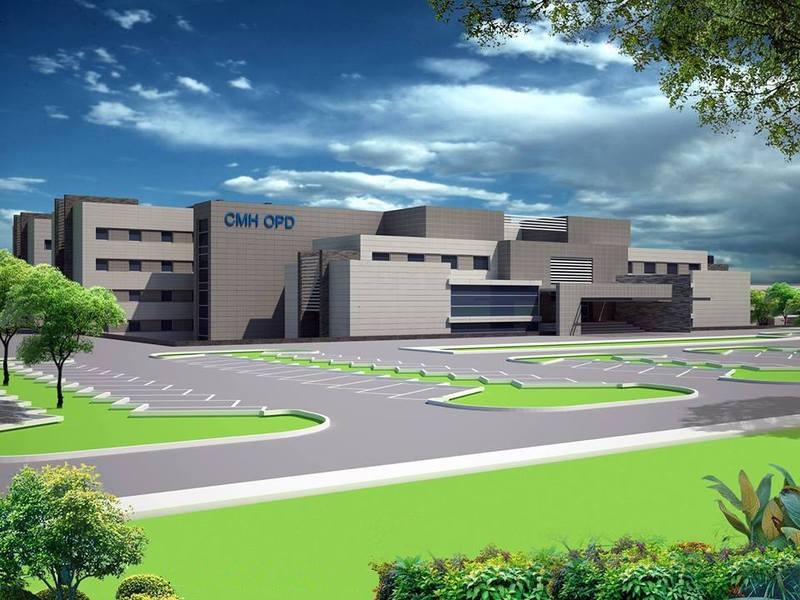

Supplement: Supplemental Information 3 [file peerj-cs-11-2980-s003.zip › cs-106973-Project_code_updated/supplemental/cs-106973-Project_code/Project code/try1/media/cmh.jpg]

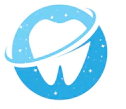

Supplement: Supplemental Information 3 [file peerj-cs-11-2980-s003.zip › cs-106973-Project_code_updated/supplemental/cs-106973-Project_code/Project code/try1/media/dentist.PNG]

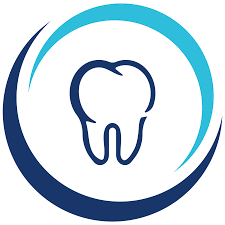

Supplement: Supplemental Information 3 [file peerj-cs-11-2980-s003.zip › cs-106973-Project_code_updated/supplemental/cs-106973-Project_code/Project code/try1/media/dentistry.jpg]

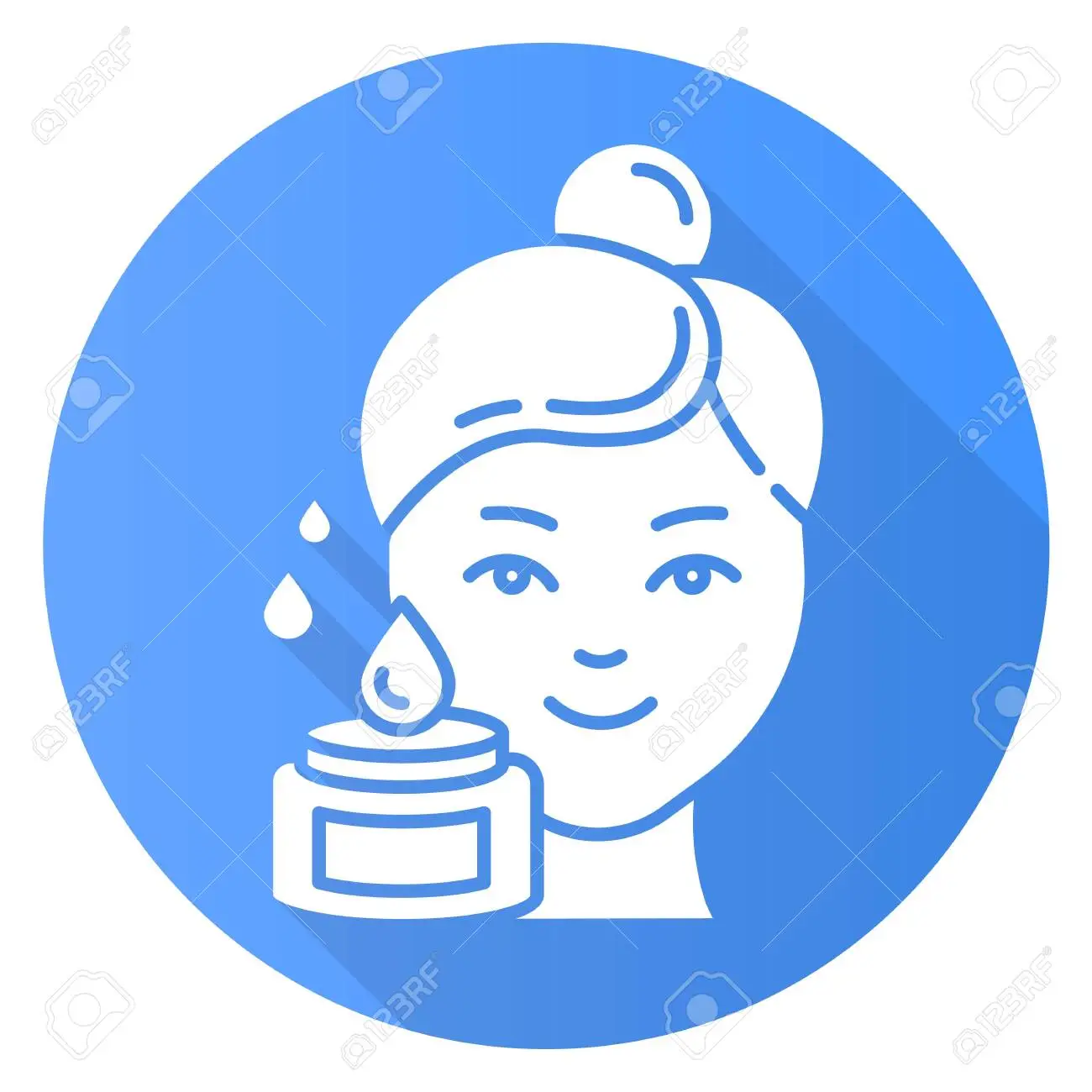

Supplement: Supplemental Information 3 [file peerj-cs-11-2980-s003.zip › cs-106973-Project_code_updated/supplemental/cs-106973-Project_code/Project code/try1/media/derma.jpg]

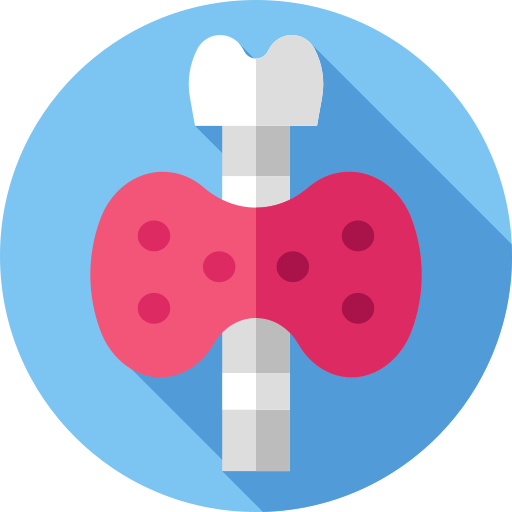

Supplement: Supplemental Information 3 [file peerj-cs-11-2980-s003.zip › cs-106973-Project_code_updated/supplemental/cs-106973-Project_code/Project code/try1/media/endo.jpg]

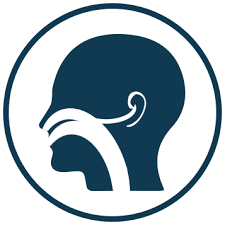

Supplement: Supplemental Information 3 [file peerj-cs-11-2980-s003.zip › cs-106973-Project_code_updated/supplemental/cs-106973-Project_code/Project code/try1/media/ent.jpg]

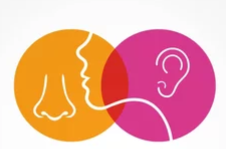

Supplement: Supplemental Information 3 [file peerj-cs-11-2980-s003.zip › cs-106973-Project_code_updated/supplemental/cs-106973-Project_code/Project code/try1/media/ENT.PNG]

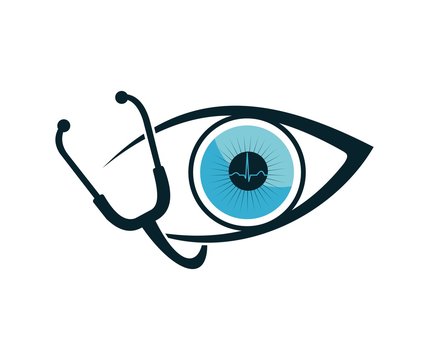

Supplement: Supplemental Information 3 [file peerj-cs-11-2980-s003.zip › cs-106973-Project_code_updated/supplemental/cs-106973-Project_code/Project code/try1/media/eye.jpg]

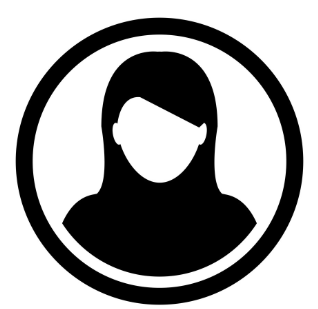

Supplement: Supplemental Information 3 [file peerj-cs-11-2980-s003.zip › cs-106973-Project_code_updated/supplemental/cs-106973-Project_code/Project code/try1/media/female.PNG]

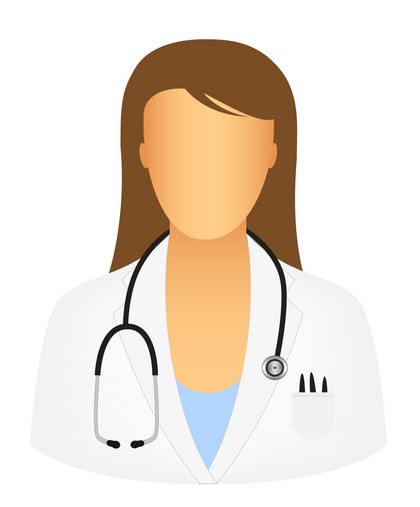

Supplement: Supplemental Information 3 [file peerj-cs-11-2980-s003.zip › cs-106973-Project_code_updated/supplemental/cs-106973-Project_code/Project code/try1/media/female1.jpg]

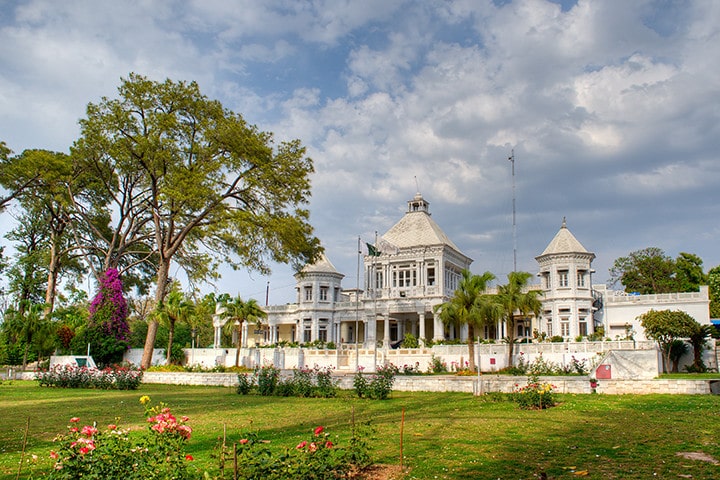

Supplement: Supplemental Information 3 [file peerj-cs-11-2980-s003.zip › cs-106973-Project_code_updated/supplemental/cs-106973-Project_code/Project code/try1/media/fjwu.jpg]

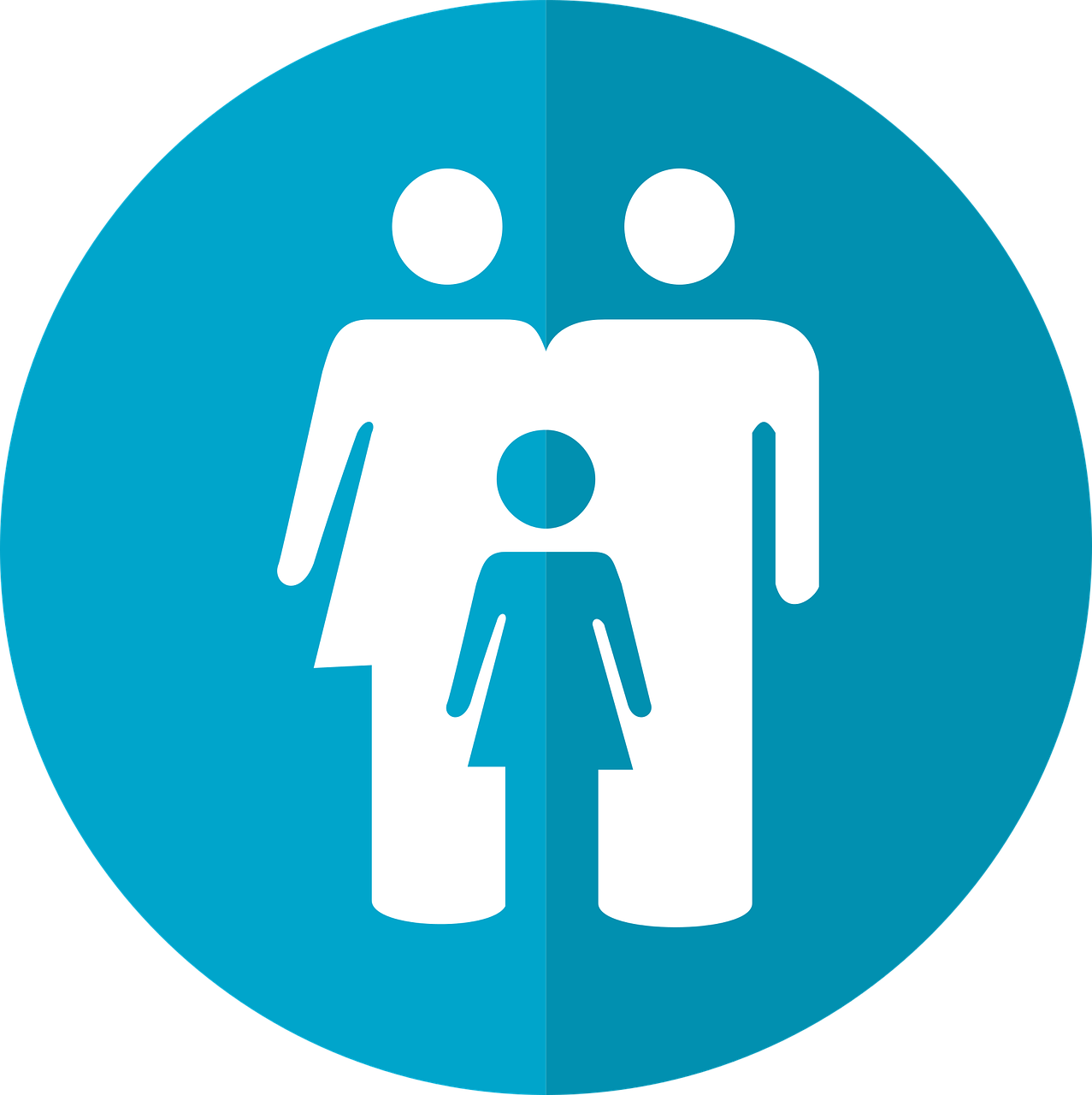

Supplement: Supplemental Information 3 [file peerj-cs-11-2980-s003.zip › cs-106973-Project_code_updated/supplemental/cs-106973-Project_code/Project code/try1/media/fm.jpg]

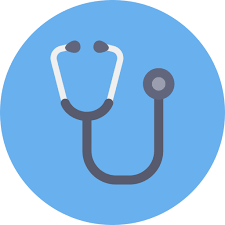

Supplement: Supplemental Information 3 [file peerj-cs-11-2980-s003.zip › cs-106973-Project_code_updated/supplemental/cs-106973-Project_code/Project code/try1/media/gm.jpg]

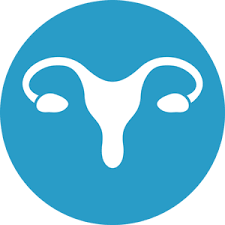

Supplement: Supplemental Information 3 [file peerj-cs-11-2980-s003.zip › cs-106973-Project_code_updated/supplemental/cs-106973-Project_code/Project code/try1/media/gn.jpg]

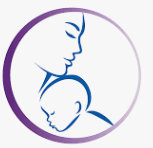

Supplement: Supplemental Information 3 [file peerj-cs-11-2980-s003.zip › cs-106973-Project_code_updated/supplemental/cs-106973-Project_code/Project code/try1/media/gynecologist.PNG]

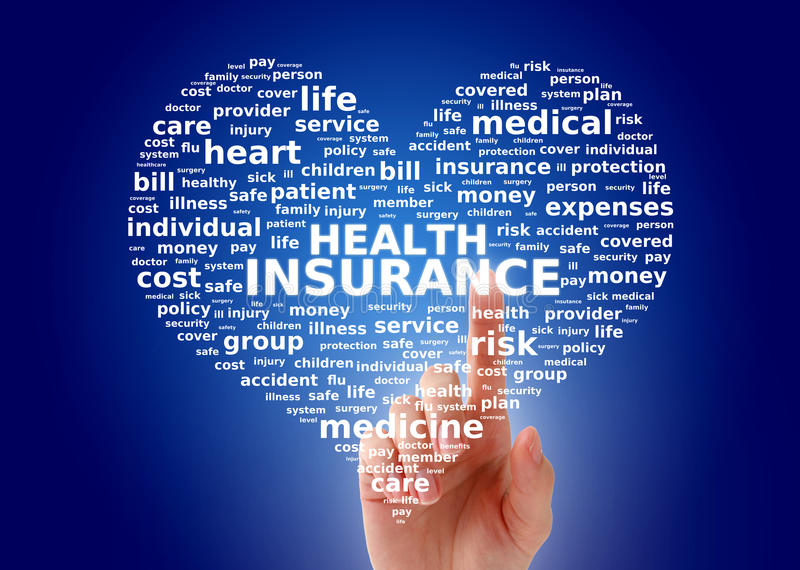

Supplement: Supplemental Information 3 [file peerj-cs-11-2980-s003.zip › cs-106973-Project_code_updated/supplemental/cs-106973-Project_code/Project code/try1/media/images3.jpg]

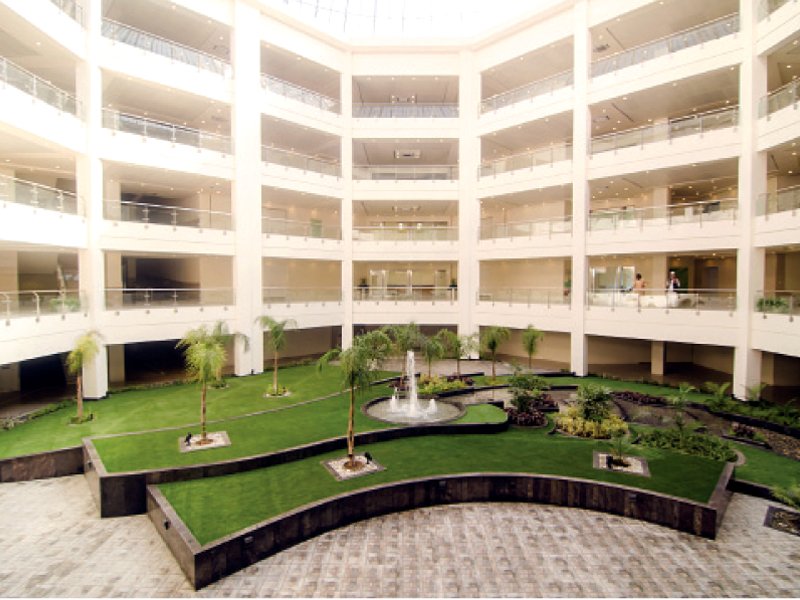

Supplement: Supplemental Information 3 [file peerj-cs-11-2980-s003.zip › cs-106973-Project_code_updated/supplemental/cs-106973-Project_code/Project code/try1/media/mh.jpg]

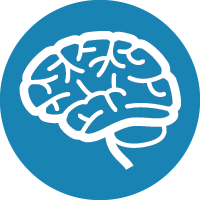

Supplement: Supplemental Information 3 [file peerj-cs-11-2980-s003.zip › cs-106973-Project_code_updated/supplemental/cs-106973-Project_code/Project code/try1/media/neuro.jpg]

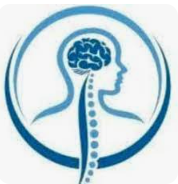

Supplement: Supplemental Information 3 [file peerj-cs-11-2980-s003.zip › cs-106973-Project_code_updated/supplemental/cs-106973-Project_code/Project code/try1/media/neuro.PNG]

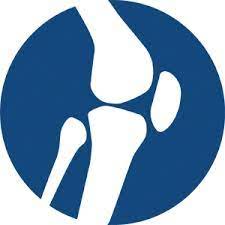

Supplement: Supplemental Information 3 [file peerj-cs-11-2980-s003.zip › cs-106973-Project_code_updated/supplemental/cs-106973-Project_code/Project code/try1/media/ortho.jpg]

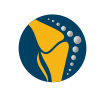

Supplement: Supplemental Information 3 [file peerj-cs-11-2980-s003.zip › cs-106973-Project_code_updated/supplemental/cs-106973-Project_code/Project code/try1/media/Orthopedic.PNG]

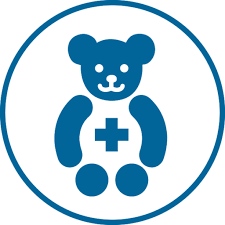

Supplement: Supplemental Information 3 [file peerj-cs-11-2980-s003.zip › cs-106973-Project_code_updated/supplemental/cs-106973-Project_code/Project code/try1/media/paed.jpg]

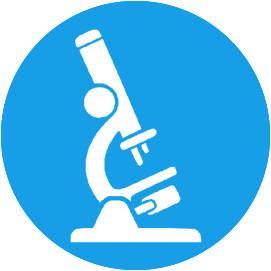

Supplement: Supplemental Information 3 [file peerj-cs-11-2980-s003.zip › cs-106973-Project_code_updated/supplemental/cs-106973-Project_code/Project code/try1/media/path.jpg]

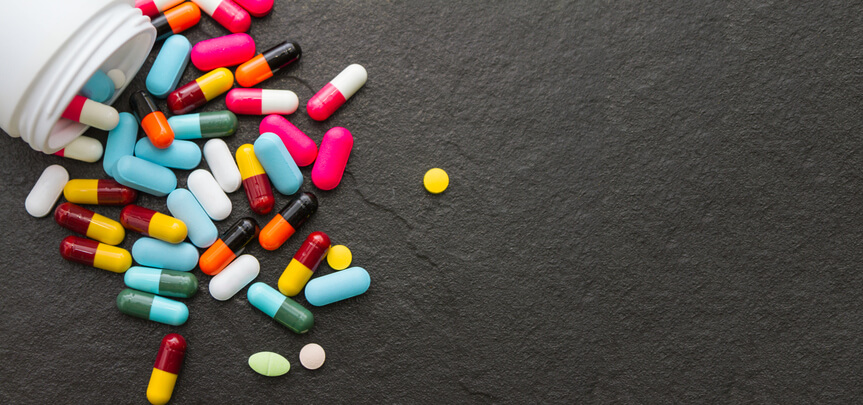

Supplement: Supplemental Information 3 [file peerj-cs-11-2980-s003.zip › cs-106973-Project_code_updated/supplemental/cs-106973-Project_code/Project code/try1/media/pill.jpeg]

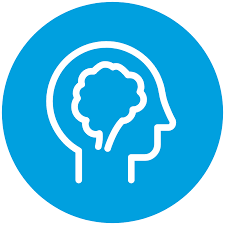

Supplement: Supplemental Information 3 [file peerj-cs-11-2980-s003.zip › cs-106973-Project_code_updated/supplemental/cs-106973-Project_code/Project code/try1/media/psy (1).jpg]

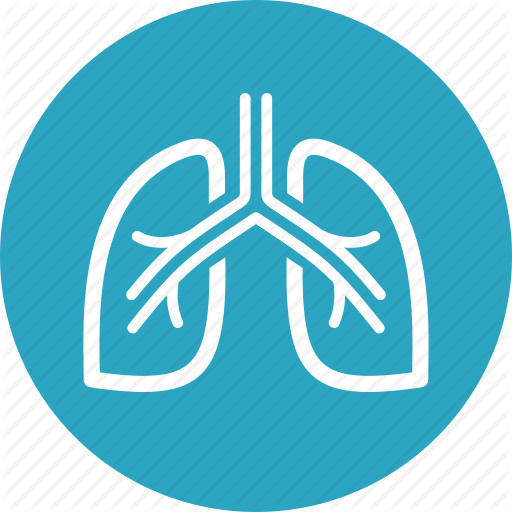

Supplement: Supplemental Information 3 [file peerj-cs-11-2980-s003.zip › cs-106973-Project_code_updated/supplemental/cs-106973-Project_code/Project code/try1/media/pulm.jpg]

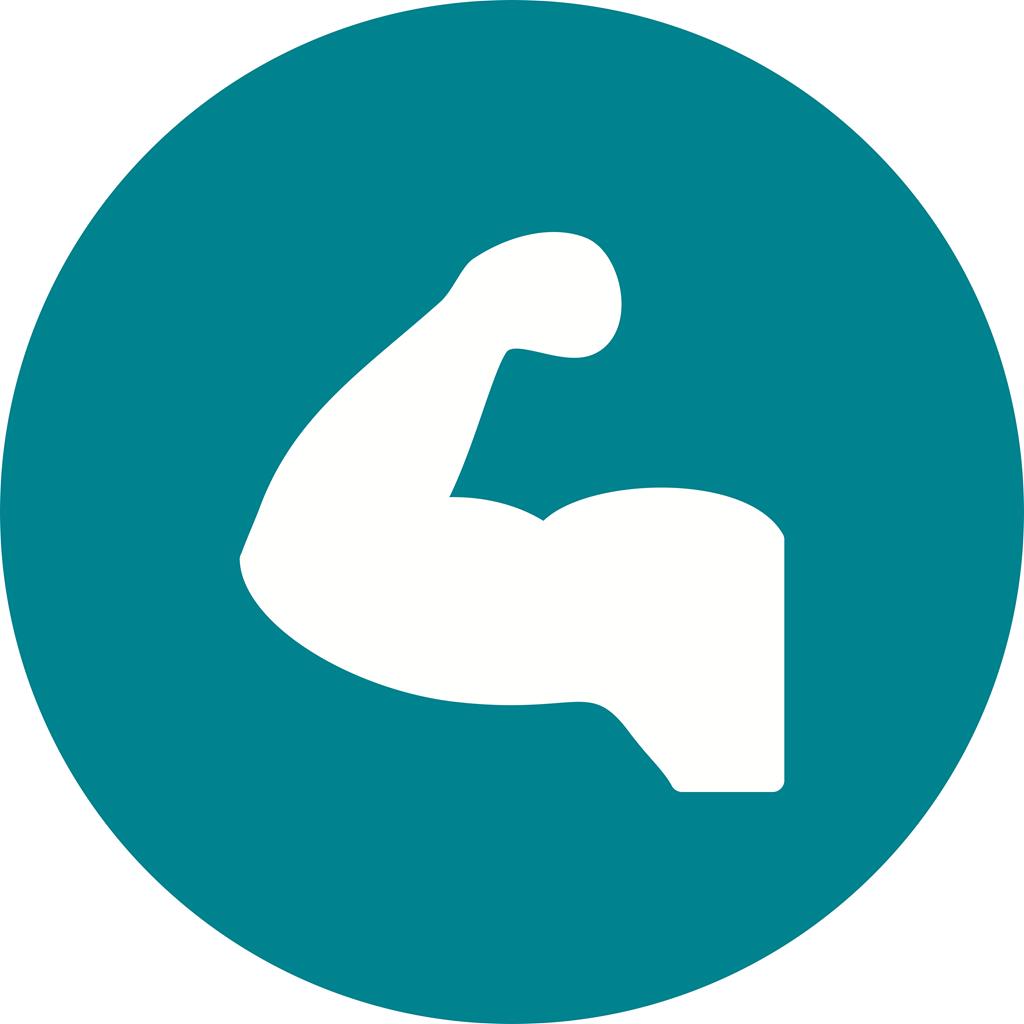

Supplement: Supplemental Information 3 [file peerj-cs-11-2980-s003.zip › cs-106973-Project_code_updated/supplemental/cs-106973-Project_code/Project code/try1/media/rheu.jpg]

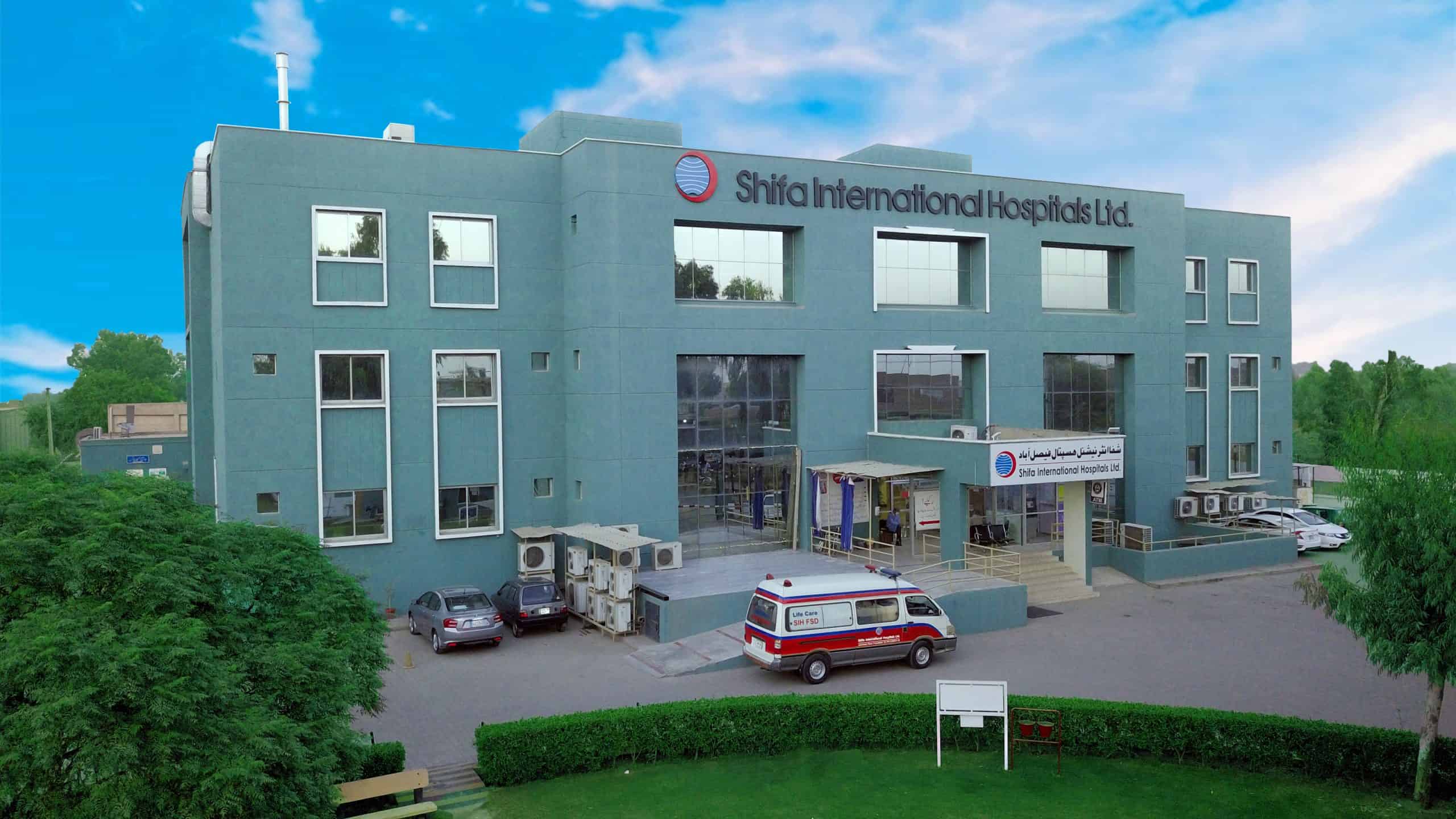

Supplement: Supplemental Information 3 [file peerj-cs-11-2980-s003.zip › cs-106973-Project_code_updated/supplemental/cs-106973-Project_code/Project code/try1/media/Shifa.jpg]

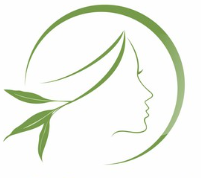

Supplement: Supplemental Information 3 [file peerj-cs-11-2980-s003.zip › cs-106973-Project_code_updated/supplemental/cs-106973-Project_code/Project code/try1/media/skin.PNG]

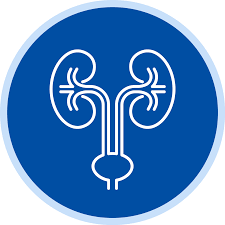

Supplement: Supplemental Information 3 [file peerj-cs-11-2980-s003.zip › cs-106973-Project_code_updated/supplemental/cs-106973-Project_code/Project code/try1/media/urology.jpg]

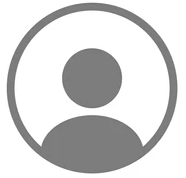

Supplement: Supplemental Information 3 [file peerj-cs-11-2980-s003.zip › cs-106973-Project_code_updated/supplemental/cs-106973-Project_code/Project code/try1/media/user.PNG]

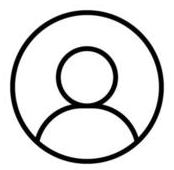

Supplement: Supplemental Information 3 [file peerj-cs-11-2980-s003.zip › cs-106973-Project_code_updated/supplemental/cs-106973-Project_code/Project code/try1/media/user1.PNG]

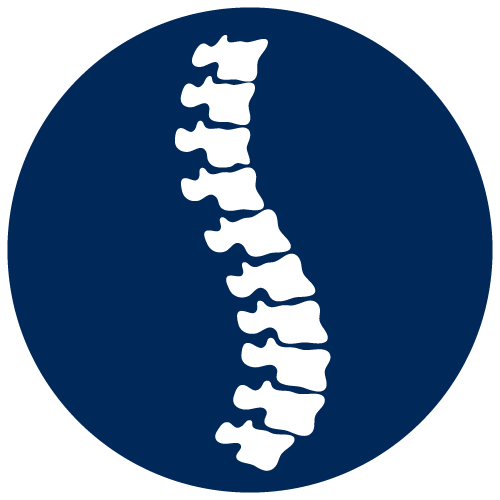

Supplement: Supplemental Information 3 [file peerj-cs-11-2980-s003.zip › cs-106973-Project_code_updated/supplemental/cs-106973-Project_code/Project code/try1/media/vs.jpg]
